# Impaired proprioception and magnified scaling of proprioceptive error responses in chronic stroke

**DOI:** 10.1186/s12984-024-01350-9

**Published:** 2024-04-09

**Authors:** Duncan Thibodeau Tulimieri, Jennifer A. Semrau

**Affiliations:** 1 Department of Kinesiology and Applied Physiology, University of Delaware, Newark, USA; 2 Program in Biomechanics and Movement Science (BIOMS), University of Delaware, 100 Discovery Blvd, Tower at STAR, Rm 234, Newark, DE 19713 USA; 3 Department of Biomedical Engineering, University of Delaware, Newark, USA

**Keywords:** Proprioception, Stroke, Robotics, Sensorimotor, Speed, Distance

## Abstract

**Background:**

Previous work has shown that ~ 50–60% of individuals have impaired proprioception after stroke. Typically, these studies have identified proprioceptive impairments using a narrow range of reference movements. While this has been important for identifying the prevalence of proprioceptive impairments, it is unknown whether these error responses are consistent for a broad range of reference movements. The objective of this study was to characterize proprioceptive accuracy as function of movement speed and distance in stroke.

**Methods:**

Stroke (N = 25) and controls (N = 21) completed a robotic proprioception test that varied movement speed and distance. Participants mirror-matched various reference movement speeds (0.1–0.4 m/s) and distances (7.5–17.5 cm). Spatial and temporal parameters known to quantify proprioception were used to determine group differences in proprioceptive accuracy, and whether patterns of proprioceptive error were consistent across testing conditions within and across groups.

**Results:**

Overall, we found that stroke participants had impaired proprioception compared to controls. Proprioceptive errors related to tested reference movement scaled similarly to controls, but some errors showed amplified scaling (e.g., significantly overshooting or undershooting reference speed). Further, interaction effects were present for speed and distance reference combinations at the extremes of the testing distribution.

**Conclusions:**

We found that stroke participants have impaired proprioception and that some proprioceptive errors were dependent on characteristics of the movement (e.g., speed) and that reference movements at the extremes of the testing distribution resulted in significantly larger proprioceptive errors for the stroke group. Understanding how sensory information is utilized across a broad spectrum of movements after stroke may aid design of rehabilitation programs.

## Background

Proprioception, the sense of our body’s location and motion in space [1], is necessary for coordinated movement. Our group and others have detailed that proprioception is impaired in ~ 30–60% of individuals after stroke [2–9]. Loss or impairment of proprioception can result in poor coordination [10], decreased stability and interlimb coordination [11, 12], reduced motor learning [13], and overall poorer function and independence [14]. While the functional implications for stroke survivors with proprioceptive impairments are broad, our understanding of the scope and impact of these impairments to movement is limited.

To date, a variety of techniques have been employed to identify deficits in position sense and kinesthesia of the wrist [2, 15] or the limb [4, 6, 16–23]. Here, the experimenter or device passively moves the participant’s hand or limb to a pre-determined location. The participant then indicates using a protractor or other representational map of space (i.e., opposite limb), where they feel that their wrist or limb is located. While these techniques have been successful in identifying proprioceptive impairments, these paradigms have been limited in that they only observed a single type of movement or minimal variations (e.g., different angles) on a single type of movement [4, 6, 16, 18, 22, 24, 25]. Therefore, these paradigms fail to broadly survey movements similarly to how they are produced in everyday functional activities.

The contributions of proprioception to movement execution are complex and include several factors that can contribute to changes in proprioceptive error detection and accuracy outside of the impact of stroke, including limitations of the sensitivity and detection thresholds of muscle spindles [26–28], sensory attenuation [29–31], and perceptual differences in peripersonal vs. extrapersonal space [32–34]. It is likely that stroke further magnifies these systemic differences and limitations; however, we have only just begun to identify and understand the proprioceptive contributions to movement execution in stroke. While current research has demonstrated proprioceptive impairment in stroke, these paradigms have limited insights across a broad range of movements, especially when we consider the broad ranging neural damage that can impact proprioception [35, 36]. Therefore, the main goal of the current study was to examine how stroke affects proprioceptive error to reference movements drawn from broad speed and distance distributions within the upper limbs. Reference movements are defined as passive movements of the more affected limb that were matched. We predicted that individuals with chronic stroke will have greater proprioceptive impairments compared to age-matched controls and that these impairments would be most salient for reference movements drawn from both tails of the distributions (e.g., slower or longer movements) [37]. By identifying proprioceptive error to a broad range of reference movement characteristics, we can better understand how upper limb proprioceptive accuracy contributes to impairments in sensorimotor function and subsequently inform rehabilitation practices.

## Methods

We recruited 25 individuals with chronic stroke and 21 age-matched controls to participate in this study. The following inclusion criteria were used for all participants: normal or corrected-to-normal vision and ≥ 18 years old. Control participants were age-matched to participants with stroke. The following exclusion criteria were used for control participants: history of disease impacting sensation (e.g., diabetic neuropathy), neurological disease or injury (e.g., Parkinson’s Disease), or upper body injury affecting the limb (e.g., rotator cuff tear). The following exclusion were used for participants with stroke: multiple and/or bilateral stroke, aphasia, neurological disease or injury other than stroke, moderate to severe cognitive impairment [Montreal Cognitive Assessment (MoCA) [38] score ≤ 17], or upper body injury affecting the upper limb. Handedness was determined using self-report and the Edinburgh Handedness Inventory [39]. The study received approval from the University of Delaware Institutional Review Board. All participants provided written informed consent prior to participation. Data included in the currently study for the control group has been previously reported on in a recent manuscript by our group [37].

### Experimental apparatus

**Fig. 1:**
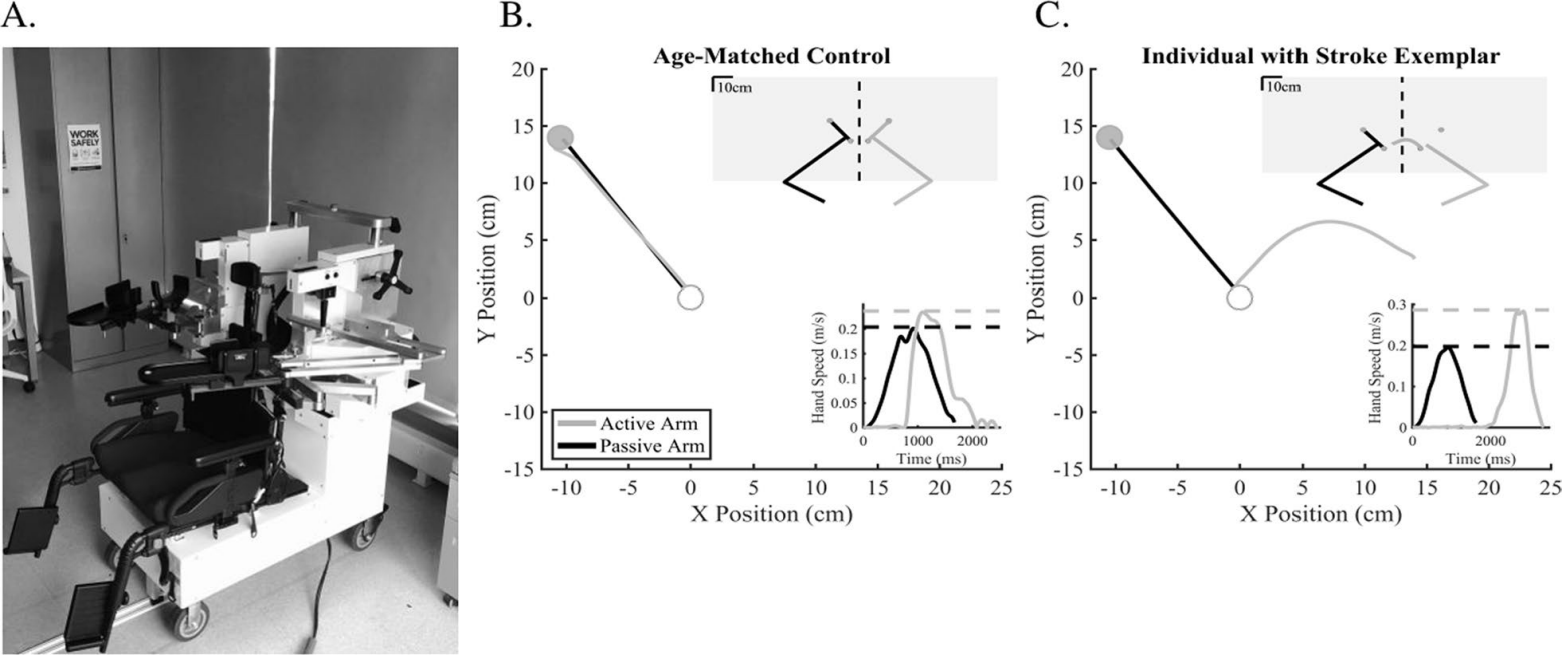
**A** KINARM exoskeleton robot. **B**, **C** Hand position data (large graph) with insets of hand speed (bottom right) and total workspace overhead view (upper right). In **B** and **C**, the white target indicates the start target and the gray target indicates the end target. **B** Exemplar age-matched control participant with low Initial Direction Error (IDE = 2.57°), near matched Peak Speed Ratio (PSR = 0.99), low End Point Error (EPE = 1.12 cm), and short Response Latency (RL = 352 ms). **C** Exemplar individual with stroke (left side of the body is more affected, right side of the body is less affected) behavior demonstrates significantly more error across all parameters compared to the control participant (IDE = 92.7°, PSR = 1.45, EPE = 26.56 cm, RL = 1986 ms)

The KINARM Exoskeleton (BKIN Technologies, Kingston, Canada) was used to collect kinematics from the upper limbs (Fig. 1A) [40]. Participants were seated in the exoskeleton with their shoulders supported at ~ 85° abduction throughout the entire experiment. The KINARM allows for and captures movement of the arm in the upper limb as well as delivering mechanical loads to the elbow and/or shoulder. The distal and proximal segments of the robot were adjusted to match each participants’ limb length. Vision of the limbs was occluded with a metal shutter and a bib that draped the participant’s upper body.

### Experimental protocol

The task required participants to mirror-match the speed, length, and direction of a passive movement made by the robot using their opposite arm. At trial start, all visual information was extinguished, and the robot passively moved the limb by moving the arm to a randomized location. The participant then mirror-matched the movement of the robot, as it occurred, with their opposite arm (active arm). The participant then gave the experimenter verbal confirmation that they matched the robotic movement, and the experimenter pressed a key to end the trial. Once verbal confirmation was received, mirrored visual target locations of the end position (2 cm cyan circle) and fingertip cursor (1 cm white circle) feedback were displayed. Participants were instructed to move both fingertip cursors into the targets to begin the next trial. To begin the next trial, all visual information was extinguished, and the robot began the next passive movement after a randomized amount of time (400–1000 ms). The robot passively moved the *more affected* limb in the stroke group and the participant actively moved the *less affected limb.* The passively moved limb was counterbalanced in the age-matched control group (dominant arm: N = 10 and non-dominant arm: N = 11) (Table 1).

**Table 1:** Participant demographics

	Stroke (N = 25)
Age	65.40 ± 12.32
Sex	17 M, 8 F
Dominant hand	18 R, 6 L, 1 A
Side of body affected	12 R, 13 L
Dominant side more affected	N = 6
Months post stroke	60 [17, 198]
FMA-UE (maximum = 66)	60 [11, 66]
FIM (maximum = 126)	124 [105, 126]
TLT {0, 1, 2, 3}	20, 3, 2, 0
PPB	6.50 [0, 13]
BIT-C (maximum = 146)	140.60 ± 7.65
Field cut	N = 11
MoCA (maximum = 30)	25.36 ± 3.28

Values presented as either mean ± standard deviation or median [minimum, maximum]

*FMA-UE* Fugl-Meyer Assessment [Upper Extremity], *FIM* Functional Independence Measure, *TLT* Thumb Localizer Test, *PPB* Purdue Pegboard, *BIT-C* Behavioral Inattention Test, *MoCA* Montreal Cognitive Assessment

During the task, participants experienced a broad distribution of reference movement characteristics. Five reference movement distances [7.5, 10, 12.5, 15, 17.5 cm] and seven reference movement peak speeds were used [0.10, 0.15, 0.20, 0.25, 0.30, 0.35, 0.40 m/s]. Each passive robotic movement used a selected distance and speed from each distribution, with each distance-speed combination randomly repeated 10 times for a total of 350 movements. For example, a movement of 7.5 cm with a peak speed of 0.2 m/s was experienced 10 times throughout testing. The reference movement characteristics were chosen based on the range of typical day-to-day upper limb movements, as well as the capabilities of the robot. The schedule of reference movements was created to ensure that (1) each reference speed and distance was sampled equally, (2) the movement occurred in a random direction sampled from a random distribution from 0° to 360°, and (3) each distance and speed combination for the reference movement was randomly ordered. The resulting schedule was used for all participants.

### Data analysis

All metrics assessing proprioception were calculated within the period from movement onset of the passive limb to movement offset of the active limb. For passive movements generated by the robot, movement onset was defined as the time where robot hand speed exceeded 10% of peak hand speed for 50 consecutive milliseconds. For active movements generated by the participant, active movement onset was defined identically, but within the active hand speed time series. If active movement onset occurred less than 150 ms after the robot began moving the passive limb, the trial was discarded due to being anticipatory in nature (Mean ± SD, Controls: 1.1 ± 1.3 trials, Stroke: 2.1 ± 4.8 trials). Movement offset was defined as the time when the hand speed decreased below 0.01 m/s for 500 consecutive ms or when end-of-trial key press occurred, whichever occurred first. Peak speed was defined as the maximum hand speed between movement onset and movement offset.

The following spatial and temporal measures have been previously found to accurately identify proprioceptive impairments in individuals with stroke [4, 6]. For spatial measures, the data from the active arm was reflected across the x-axis to make comparisons within the same space. End Point Error (EPE) was calculated as the Euclidean distance between the active hand and the passive hand at their respective movement offset locations (Eq. 1). A larger EPE indicated a larger amount of proprioceptive error.

1 $$EPE= \sqrt{\sum {\left({Offset}_{passive} - {Offset}_{active}\right)}^{2}}.$$

Initial Direction Error (IDE) was calculated as the angle between the passive and active limb at their respective peak speeds (Eq. 2). A larger IDE indicated a larger amount of proprioceptive error, and a smaller IDE indicated a smaller amount of proprioceptive error.

2 $${IDE= cos}^{-1} \left(\frac{{Initial\; Movement\; Vector}_{passive}}{{Initial\; Movement\; Vector}_{active}}\right).$$

Path Length Ratio (PLR) was calculated as the quotient of the arc length of the passive limb and the arc length of the active limb (Eq. 3). A PLR of 1 indicated perfect path length matching, and values > 1 indicated that the length of the movement of the active arm was longer than the length of the movement for the passive arm.

3 $$PLR= \frac{{Movement\; Arc\; Length}_{active}}{{Movement\; Arc\; Length}_{passive}}.$$

Response Latency (RL) was calculated as the time difference between the movement onset of the passive arm and the movement onset of the active arm (Eq. 4). A larger Response Latency indicated it took longer for a participant to respond to the passive movement.

4 $$RL={Onset}_{active}- {Onset}_{passive}.$$

Peak Speed Ratio (PSR) was calculated as the quotient of the peak speed of the passive limb to peak speed of the active limb (Eq. 5). A PSR of 1 indicated perfect peak speed matching and values > 1 indicated the active arm had a larger peak speed than the passive arm.

5 $$PSR=\frac{{Peak\; Speed }_{active}}{{Peak\; Speed}_{passive}}.$$

### Statistical analyses

For each participant, averages were computed across movements made within each speed or distance value for each of the above-described parameters. First, we determined whether there were group (stroke vs. control) level differences within each of the tested speeds and/or distances. Here, we used permutation tests with 1,000,000 iterations and quantified magnitude of difference in performance using common language effect size (CLES) [41]. Directional permutation tests $({H}_{0}{:}\;stroke < controls)$ were used for one-sided, non-ratio measures (EPE, RL, and IDE), and non-directional permutation tests were used for two-sided, ratio measures (PLR, PSR). This analysis was repeated for a secondary analysis in which we compared within the stroke group (left-affected vs. right-affected) with only non-directional tests. The more-affected side was determined from clinical measures. Additionally, we used a non-directional permutation test to compare ages of our two groups. Second, we aimed to examine the pattern and scaling of errors across (1) speed of the reference movement, and (2) distance of the reference movement. Here, for each participant, average values from each reference speed or distance value were computed and fit to a line using ordinary least squares. For example, for each parameter, a participant would have a single average for each of the five tested reference distances, for a total of five values (e.g., average EPE values at 7.5, 10, 12.5, 15, and 17.5 cm). These values were fit to a line using ordinary least squares resulting in the calculation of intercept (β_0_) and slope (β_1_) coefficients for each participant, with the intercept representing the amount of error at 0 and the slope representing the scaling of error across reference speed or distance. Group comparisons (control vs. stroke) using the resultant coefficient values were computed using the above-described permutation test methods for β_0_ and β_1_ to determine group-level differences in magnitude and scaling of error, respectively. Lastly, we aimed to examine interaction effects of reference distance x speed within each group to determine if proprioceptive error was dependent upon both the reference speed and distance. A two-way ANOVA (distance × speed) was computed for each group, where interaction terms were calculated from average within group performance for each of the reference movement characteristics. All alpha levels were set to 0.05 and all analyses and figures were created using custom MATLAB (Mathworks, Natick, MA) scripts.

## Results

We collected data from 46 participants and compared behavior from age-matched controls and stroke participants to determine whether characteristics of the reference movement impacted proprioceptive error. No significant differences were found for age between the groups (p = 0.4, CLES = 57.33%; Control: 62.62 ± 10.61 yrs, 9 M/12 F, Stroke: 65.40 ± 12.32 yrs, 17 M/8 F). All (N = 21) age-matched controls were right-handed and N = 18 stroke participants were right-handed (N = 6 left-handed, N = 1 ambidextrous, Table 1).

### Clinical differences between individuals with right/left side more affected

To determine if there were differences between participants who had a stroke that mostly affected their right side versus participants who had a stroke that mostly affected their left side, we split up our individuals with stroke by side more affected and compared their clinical measures [Montreal Cognitive Assessment (MoCA): right-affected: 24 ± 3.7 and left-affected: 26.6 ± 2.4; p = 0.05, CLES = 70.83%; Functional Independence Measurement (FIM): right-affected: 123.8 ± 2.3 and left-affected: 121.6 ± 6.4; p = 0.34, CLES = 52.88%; Fugl-Meyer Assessment (FMA): right-affected: 57.8 ± 13.9 and left-affected: 48.1 ± 18.4; p = 0.149, CLES = 72.12%; Purdue Pegboard (PPB): right-affected: 7.0 ± 4.6 and left-affected: 3.7 ± 4.0; p = 0.071, CLES = 73.40%; Behavioral Inattention Test (BIT): right-affected: 142.0 ± 6.9 and left-affected: 139.3 ± 8.4; p = 0.434, CLES = 72.44%]. Therefore, we decided to collapse across side more affected for individuals with stroke for our main analyses but separate for secondary analyses.

### Proprioceptive error as a function of movement speed

**Fig. 2:**
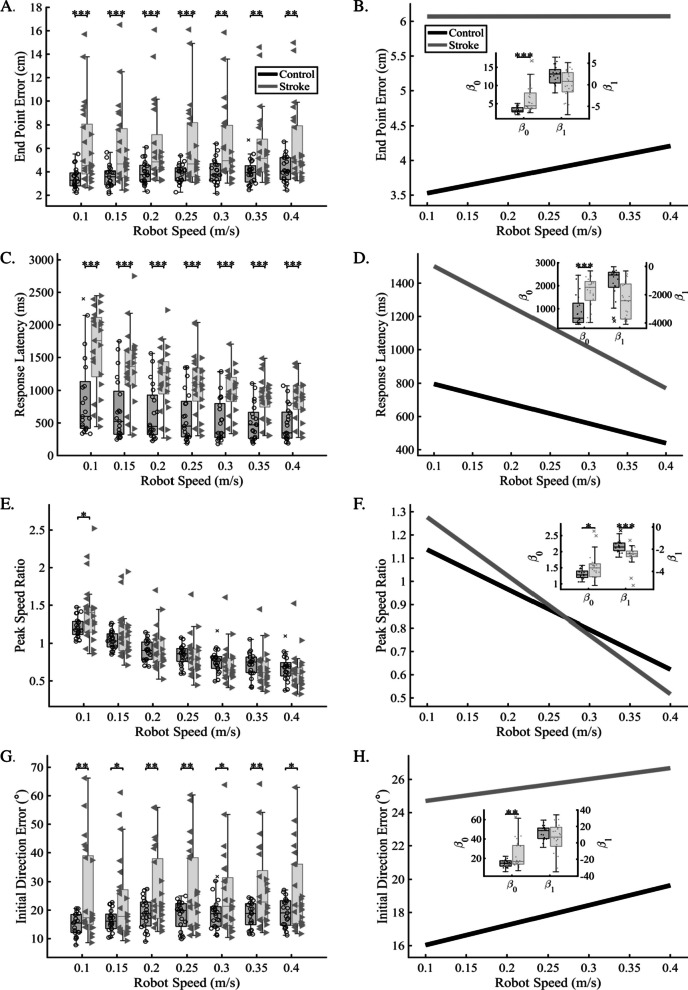
Proprioceptive accuracy at each tested robot speed. In the left column (**A**, **C**, **E**, **G**), the box plots display proprioceptive error in each of four parameters (EPE, RL, PSR, IDE) for age-matched controls (black) and individuals with stroke (gray). For stroke participants, left-facing triangles indicate individual stroke participants with the left side of the body as their more affected side, and right-facing triangles indicate individuals with the right side of the body as their more affected side. We observed that across each of the parameters in the left column that stroke participants showed significantly more error compared to age-matched controls at each of the tested robot speeds. In the right column (**B**, **D**, **F**, **H**) data was fit using ordinary least squares for individual participants to determine the intercept (${\beta }_{0}$) and slope (${\beta }_{1}$) of the average resultant behavior within groups. Here, the goal was to examine changes in pattern and/or magnitude of proprioceptive error as a function of robot speed. Solid lines indicate the bootstrapped average fit for age-matched controls (black) and individuals with stroke (gray). Insets indicate fit coefficients (${\beta }_{0}$ and ${\beta }_{1}$), with individual participant coefficient data included on the box plot. Overall, we found that for the parameters tested, the pattern (slope) was similar between age-matched controls and individuals with stroke, but that the magnitude (intercept) was significantly different for all individuals with stroke suggesting that while stroke increases the overall magnitude, the pattern of error response is preserved when the speed of the reference movement is changed. *p ≤ 0.05, **p ≤ 0.01

To determine whether reference movement speeds were perceived differently by stroke participants, matching movements were grouped according to each of the reference speeds, regardless of movement distance. Initial comparisons were completed for group averages within each movement speed to determine if stroke participants had significantly impaired sense of speed compared to controls (Fig. 2, left column, A, C, E, G). We found that stroke participants had significantly increased error compared to controls for all tested reference speeds, for all parameters except PSR, which was significantly different for only the 0.1 m/s reference speed (Fig. 2, see Table 2 for full statistics). These results demonstrate that regardless of tested speed, stroke participants had significantly impaired proprioception across spatial and temporal aspects of the movement. To understand whether error changed as a function of tested speed, we fit data from each participant using ordinary least squares and then compared the group distributions for each coefficient [intercept (β_0_) and slope (β_1_)]. Overall, we found that stroke participants had significantly higher intercept values than age-matched controls for all parameters (Fig. 2: right column, B, D, F, H) [EPE (cm): Stroke = 6.1 ± 3.6, Control = 3.3 ± 0.8; p < 0.001, CLES = 78.9%; RL (ms): Stroke = 1747 ± 641, Control = 915 ± 660; p < 0.001, CLES = 81.5%; PLR: Stroke = 1.3 ± 0.3, Control = 1.1 ± 0.1; p < 0.001, CLES = 85.1%; IDE (°): Stroke = 24.0 ± 15.5, Control = 14.8 ± 3.8; p = 0.003, CLES = 64.2%; PSR: Stroke = 1.5 ± 0.4, Control = 1.3 ± 0.1; p = 0.02, CLES = 69.9%]. In contrast, we found that for stroke participants, PSR was the only parameter with a significantly different slope (β_1_) value than controls (PSR: Stroke = − 2.5 ± 0.9, Control = − 1.7 ± 0.6; p < 0.001, CLES = 81.9%). Overall, we see that the magnitude of proprioceptive impairment is elevated across all reference speeds in the majority of parameters for stroke participants, while the error response for matching the speed of the movement (PSR) scales differently for stroke survivors where they significantly overestimate the reference movement speed at slower speeds and significantly underestimate the reference movement speed at faster speeds (Fig. 2F).

**Table 2:** Statistics for within speed and distance comparisons between age-matched controls and individuals with stroke

	Parameter				
	End point error (cm)	Initial direction error (°)	Path length ratio	Response latency (ms)	Peak speed ratio
Speed (m/s)					
0.10	CLES = 80.38; p < 0.001***	CLES = 69.14; p = 0.001**	CLES = 79.43; p = 0.002**	CLES = 81.14; p < 0.001***	CLES = 69.33; p = 0.025*
0.15	CLES = 76.57; p < 0.001***	CLES = 59.43; p = 0.020*	CLES = 84.00; p < 0.001***	CLES = 80.95; p < 0.001***	CLES = 54.86; p = 0.205
0.20	CLES = 77.14; p < 0.001***	CLES = 64.95; p = 0.006**	CLES = 76.38; p = 0.002**	CLES = 80.76; p < 0.001***	CLES = 54.10; p = 0.847
0.25	CLES = 72.00; p = 0.001***	CLES = 66.10; p = 0.006**	CLES = 73.14; p = 0.003**	CLES = 80.38; p < 0.001***	CLES = 58.48; p = 0.766
0.30	CLES = 72.38; p = 0.001**	CLES = 62.10; p = 0.027*	CLES = 70.67; p = 0.007**	CLES = 83.05; p < 0.001***	CLES = 63.43; p = 0.456
0.35	CLES = 74.10; p = 0.001**	CLES = 70.10; p = 0.002**	CLES = 65.90; p = 0.027*	CLES = 80.95; p < 0.001***	CLES = 65.71; p = 0.279
0.40	CLES = 69.33; p = 0.003**	CLES = 61.71; p = 0.015*	CLES = 65.90; p = 0.039*	CLES = 84.19; p < 0.001***	CLES = 64.38; p = 0.391
Distance (cm)					
7.5	CLES = 73.71; p < 0.001***	CLES = 65.71; p = 0.006**	CLES = 71.24; p = 0.007**	CLES = 84.95; p < 0.001***	CLES = 55.43; p = 0.912
10.0	CLES = 76.38; p < 0.001***	CLES = 66.10; p = 0.007**	CLES = 72.95; p = 0.003**	CLES = 81.90; p < 0.001***	CLES = 53.90; p = 0.830
12.5	CLES = 78.29; p < 0.001***	CLES = 66.29; p = 0.005**	CLES = 75.24; p = 0.001**	CLES = 82.48; p < 0.001***	CLES = 53.52; p = 0.779
15.0	CLES = 74.29; p = 0.001***	CLES = 62.10; p = 0.010**	CLES = 78.10; p = 0.005**	CLES = 80.19; p < 0.001***	CLES = 53.71; p = 0.829
17.5	CLES = 71.62; p = 0.002**	CLES = 67.62; p = 0.004**	CLES = 73.90; p = 0.012*	CLES = 80.19; p < 0.001***	CLES = 50.10; p = 0.512

*CLES* common language effect size

Significance level: *p ≤ 0.05, **p ≤ 0.01, ***p ≤ 0.001

We completed secondary analyses to examine whether there were significant differences within the stroke group for participants who were left-affected vs. right-affected. When we examined parameter error (e.g., EPE) as a function of reference speed, we found that those that were left-affected had significantly more error than right-affected subjects for EPE and IDE at each tested reference speed (Table 3). For PLR, those that were left-affected had significantly higher error for only the 0.25 m/s reference speed. These results are supported by significantly higher intercept (β_0_) values for EPE and IDE for left-affected individuals compared to right-affected.

**Table 3:** Robot speed analysis statistics for comparisons between right more-affected and left more-affected for individuals with stroke

	Parameter				
	EPE (cm)	IDE (°)	PLR	RL (ms)	PSR
Speed (m/s)					
0.10	R: 4.3 ± 1.2 L: 7.8 ± 4.0 CLES = 76.9; p = 0.006**	R: 16.7 ± 7.2 L: 33.3 ± 17.6 CLES = 80.8; p = 0.005**	R: 1.2 ± 0.2 L: 1.3 ± 0.3 CLES = 60.3; p = 0.260	R: 1571 ± 704 L: 1712 ± 548 CLES = 54.5; p = 0.577	R: 1.4 ± 0.4 L: 1.5 ± 0.3 CLES = 62.8; p = 0.506
0.15	R: 4.2 ± 1.0 L: 7.6 ± 3.9 CLES = 76.9; p = 0.004**	R: 15.5 ± 4.1 L: 29.7 ± 16.3 CLES = 78.8; p = 0.005**	R: 1.2 ± 0.2 L: 1.3 ± 0.3 CLES = 69.2; p = 0.141	R: 1339 ± 647 L: 1312 ± 457 CLES = 53.2; p = 0.908	R: 1.1 ± 0.3 L: 1.2 ± 0.3 CLES = 62.8; p = 0.329
0.20	R: 4.4 ± 0.9 L: 7.9 ± 4.0 CLES = 76.3; p = 0.006**	R: 19.6 ± 5.2 L: 33.3 ± 15.4 CLES = 75.0; p = 0.008**	R: 1.1 ± 0.2 L: 1.3 ± 0.3 CLES = 67.3; p = 0.108	R: 1129 ± 546 L: 1225 ± 374 CLES = 58.3; p = 0.614	R: 0.9 ± 0.2 L: 1.0 ± 0.3 CLES = 58.3; p = 0.398
0.25	R: 4.2 ± 0.9 L: 7.7 ± 4.2 CLES = 78.8; p = 0.004**	R: 19.1 ± 5.7 L: 33.2 ± 16.9 CLES = 70.5; p = 0.011*	R: 1.1 ± 0.1 L: 1.3 ± 0.4 CLES = 75.6; p = 0.020*	R: 936 ± 386 L: 1238 ± 469 CLES = 69.2; p = 0.094	R: 0.8 ± 0.2 L: 0.9 ± 0.3 CLES = 62.2; p = 0.256
0.30	R: 4.3 ± 0.9 L: 7.8 ± 3.7 CLES = 84.0; p = 0.002**	R: 16.9 ± 4.5 L: 32.8 ± 14.6 CLES = 87.8; p = 0.001***	R: 1.1 ± 0.1 L: 1.3 ± 0.4 CLES = 71.2; p = 0.068	R: 889 ± 352 L: 1017 ± 325 CLES = 59.6; p = 0.355	R: 0.7 ± 0.2 L: 0.8 ± 0.3 CLES = 58.3; p = 0.421
0.35	R: 4.4 ± 1.0 L: 7.3 ± 3.6 CLES = 76.9; p = 0.008**	R: 21.2 ± 6.1 L: 33.0 ± 16.5 CLES = 69.9; p = 0.028*	R: 1.0 ± 0.1 L: 1.2 ± 0.4 CLES = 67.3; p = 0.101	R: 789 ± 294 L: 977 ± 316 CLES = 63.5; p = 0.138	R: 0.6 ± 0.2 L: 0.7 ± 0.3 CLES = 59.6; p = 0.519
0.40	R: 4.5 ± 0.8 L: 7.6 ± 3.9 CLES = 72.4; p = 0.008**	R: 18.6 ± 4.2 L: 32.8 ± 15.8 CLES = 73.7; p = 0.005**	R: 1.0 ± 0.2 L: 1.2 ± 0.4 CLES = 69.9; p = 0.083	R: 781 ± 282 L: 955 ± 310 CLES = 63.5; p = 0.159	R: 0.6 ± 0.2 L: 0.7 ± 0.3 CLES = 59.6; p = 0.505
OLS	Intercept: R: 4.1 ± 1.2 L: 7.9 ± 4.2 CLES = 78.8; p = 0.005**	Intercept: R: 15.6 ± 6.3 L: 31.8 ± 17.5 CLES = 78.8; p = 0.005**	Intercept: R: 1.3 ± 0.2 L: 1.4 ± 0.3 CLES = 59.6; p = 0.257	Intercept: R: 1725 ± 718 L: 1767 ± 591 CLES = 53.2; p = 0.873	Intercept: R: 1.5 ± 0.4 L: 1.6 ± 0.4 CLES = 64.1; p = 0.419
	Slope: R: 0.8 ± 3.0 L: − 0.7 ± 3.7 CLES = 60.9; p = 0.286	Slope: R: 10.4 ± 17.6 L: 3.1 ± 18.8 CLES = 63.5; p = 0.330	Slope: R: − 0.7 ± 0.3 L: − 0.4 ± 0.8 CLES = 58.3; p = 0.386	Slope: R: − 2651 ± 1418 L: − 2248 ± 1133 CLES = 62.2; p = 0.433	Slope: R: − 2.5 ± 0.8 L: − 2.6 ± 0.9 CLES = 54.5; p = 0.654

*CLES* Common Language Effect Size, *EPE* End Point Error, *IDE* Initial Direction Error, *PLR* Path Length Ratio, *RL* Response Latency, *PSR* Peak Speed Ratio, *R* Right More Affected, *L* Left More Affected, *OLS* Ordinary Least Squares

Significance level: *p ≤ 0.05, **p ≤ 0.01, and ***p ≤ 0.001

### Proprioceptive error as a function of movement distance

**Fig. 3:**
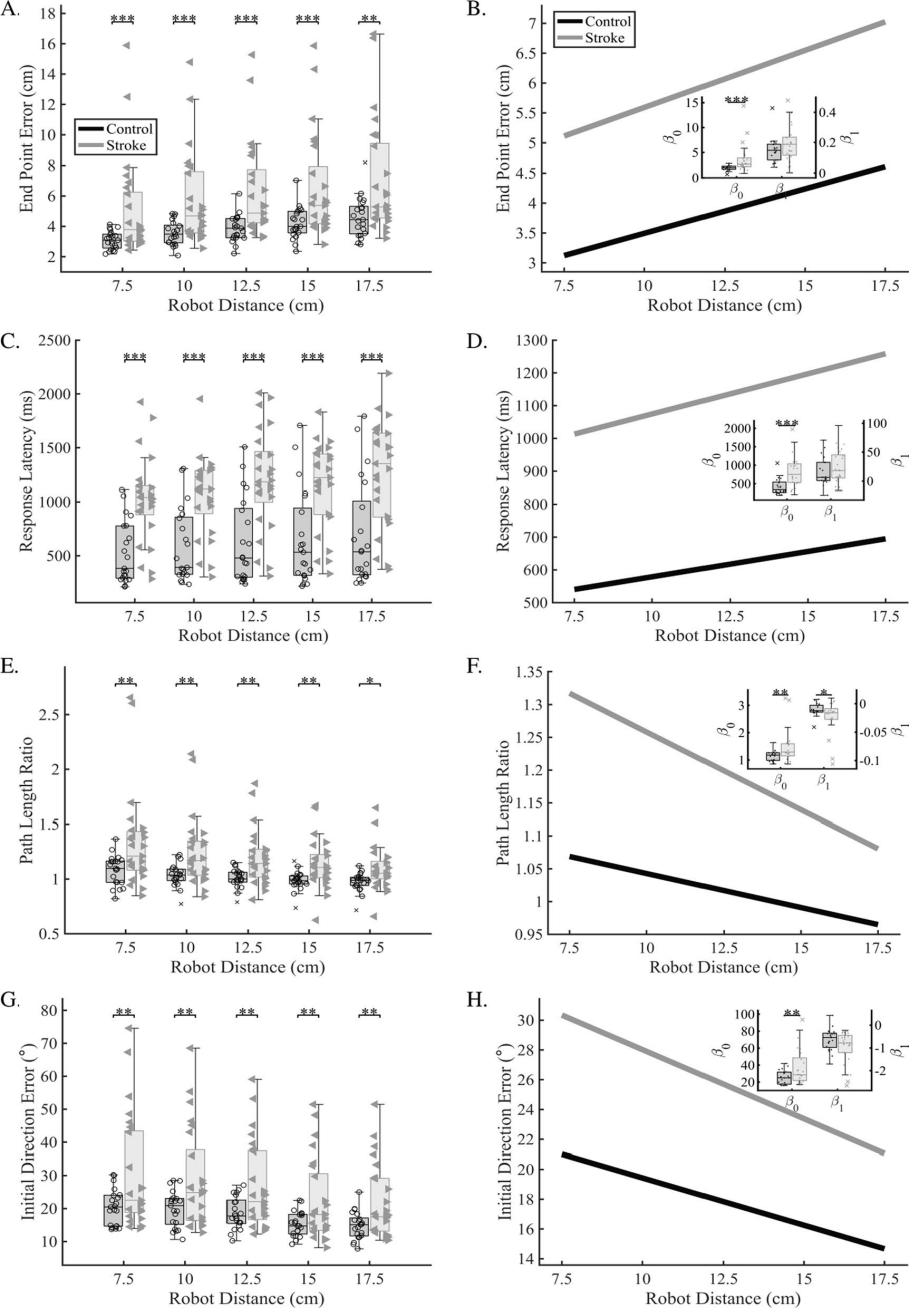
Proprioceptive accuracy at each tested robot distance. In the left column (**A**, **C**, **E**, **G**), the box plots display proprioceptive error in each of four parameters (EPE, RL, PSR, IDE) for age-matched controls (black) and individuals with stroke (gray). For stroke participants, left-facing triangles indicate individual stroke participants with the left side of the body as their more affected side, and right-facing triangles indicate individuals with the right side of the body as their more affected side. We observed that across each of the parameters in the left column, that stroke participants showed significantly more error compared to age-matched controls at each of the tested robot distances. In the right column (**B**, **D**, **F**, **H**) data was fit using ordinary least squares for individual participants to determine the intercept (${\beta }_{0}$) and slope (${\beta }_{1}$) of the average resultant behavior within participant group. Here, the goal was to examine changes in pattern and/or magnitude of proprioceptive error as a function of robot distance. Solid lines indicate the bootstrapped average fit for controls (black) and individuals with stroke (gray). Insets indicate fit coefficients (${\beta }_{0}$ and ${\beta }_{1}$), with individual participant coefficient data included on the box plot. Overall, we found that for most parameters tested, the pattern (slope) was similar between age-matched controls and individuals with stroke, but that the magnitude (intercept) was significantly different for all stroke participants suggesting that while stroke increases the overall magnitude, the pattern of error response is preserved when the distance of the reference movement is changed. Interestingly, our measure of how accurately participants matched the length of the movement (PLR, F) showed that stroke participants overestimated the distance of the passive robotic movement at shorter distances [7.5 cm, 10 cm] more than at longer distances [15 cm, 17.5 cm] compared to controls. *p ≤ 0.05, **p ≤ 0.01

We then examined whether reference movement distances were perceived differently by stroke participants. Here, matching movements were grouped according to each of the reference distances, regardless of movement speed. Within distance averages were computed for each group to determine if stroke participants had significantly impaired perception of movement distance compared to controls (Fig. 3, left column). We found that stroke participants had significantly increased error compared to controls for all tested reference distances, for all parameters except PSR, which was not significantly different from controls (see Table 2 for full statistics). These results demonstrate that regardless of reference distance, stroke participants had significantly impaired proprioception across spatial and temporal aspects of the movement, regardless of the distance of the reference movement. We aimed to determine whether proprioceptive error changed as a function of tested distance and fit data from each participant using ordinary least squares to compare the group distributions for each coefficient (β_0_ and β_1_). After fitting the data and comparing between groups, we found that stroke participants had significantly larger error magnitude across (intercept) all parameters (Fig. 3, right column) [EPE (cm): Stroke = 3.7 ± 2.9, Control = 2.0 ± 0.5; p < 0.001, CLES = 77.9%; RL (ms): Stroke = 829 ± 415, Control = 424 ± 213; p < 0.001, CLES = 81.9%; PLR: Stroke = 1.5 ± 0.6, Control = 1.1 ± 0.2; p = 0.007, CLES = 71.2%; IDE (°): Stroke = 37.3 ± 21.2, Control = 25.8 ± 8.1; p = 0.009, CLES = 65.7%], except for PSR (Stroke = 0.7 ± 0.3, Control = 0.7 ± 0.2; p = 0.68, CLES = 56.8%). In contrast, resulting fits for slope across reference distances were only significant for PLR (Stroke = − 0.02 ± 0.03, Control = − 0.01 ± 0.01; p = 0.04, CLES = 65.3%), suggesting that in addition to higher overall error, measures of matching movement length showed different scaling in stroke participants where errors were higher for shorter distances and lesser for longer distances compared to controls (Fig. 3E, F).

We completed secondary analyses to examine whether there were significant differences within the stroke group for participants who were left-affected vs. right-affected. When we examined parameter error as a function of reference distance, we found that those that were left-affected had significantly higher error compared for EPE and IDE at each tested reference distance compared to right-affected stroke, and significantly higher error for PLR for only the 10 cm reference distance. When we examined the overall level error and scaling across distances, we observed that left-affected individuals had significantly higher intercept (β_0_) values for EPE (right-affected = 2.4 ± 0.5 cm, left-affected = 4.8 ± 3.6 cm; p = 0.015, CLES = 76.3%, Table 4). Additionally, we observed that when we considered performance across all reference distances, left-affected individuals had overall higher IDE that scaled differently compared to right-affected individuals, with larger IDE at shorter reference distances and smaller IDE at longer reference distances [β_0_ (°): right-affected = 25.5 ± 5.2, left-affected = 48.2 ± 24.7; p = 0.004, CLES = 75.6%; β_1_ (°): right-affected = 0.6 ± 0.2, left-affected = − 1.3 ± 0.8; p = 0.010, CLES = 73.1%, Table 4]. We observed a similar difference for PLR and PSR, where left-affected stroke had significantly more error, and significantly different slopes indicating different scaling of errors. For PLR, we observed that left-affected stroke overshot the reference distance at short distances and converged with right-affected stroke at the longest reference distances. For PSR, we observed that left-affected stroke had a significantly flatter slope than those with right-affected stroke, suggesting a flattening of the scaled response (PLR: β_0_: right-affected = 1.2 ± 0.3, left-affected = 1.7 ± 0.7; p = 0.021, CLES = 74.4%; β_1_: right-affected = − 0.01 ± 0.01, left-affected = − 0.04 ± 0.03; p = 0.015, CLES = 76.9%; PSR: β_0_: right-affected = 0.5 ± 0.2, left-affected = 0.8 ± 0.3; p = 0.042, CLES = 75.0%; β_1_: right-affected = 0.02 ± 0.01, left-affected = 0.01 ± 0.01; p = 0.009, CLES = 78.8%; Table 4).

**Table 4:** Robot distance analysis statistics for comparisons between right more-affected and left more-affected for individuals with stroke

	Parameter				
	EPE (cm)	IDE (°)	PLR	RL (ms)	PSR
Distance (cm)					
7.5	R: 3.4 ± 0.4 L: 6.5 ± 3.9 CLES = 75.0; p = 0.004**	R: 20.6 ± 4.2 L: 38.7 ± 19.7 CLES = 75.0; p = 0.004**	R: 1.2 ± 0.2 L: 1.5 ± 0.5 CLES = 70.5; p = 0.061	R: 948 ± 421 L: 1073 ± 383 CLES = 60.9; p = 0.447	R: 0.7 ± 0.2 L: 0.9 ± 0.3 CLES = 68.6; p = 0.188
10.0	R: 4.0 ± 0.8 L: 7.2 ± 3.5 CLES = 78.2; p = 0.004**	R: 20.3 ± 5.6 L: 36.0 ± 17.2 CLES = 75.6; p = 0.006**	R: 1.1 ± 0.2 L: 1.4 ± 0.4 CLES = 76.3; p = 0.023*	R: 950 ± 363 L: 1138 ± 364 CLES = 60.3; p = 0.211	R: 0.8 ± 0.2 L: 0.9 ± 0.3 CLES = 71.2; p = 0.191
12.5	R: 4.6 ± 0.9 L: 7.8 ± 3.7 CLES = 73.7; p = 0.004**	R: 19.2 ± 4.4 L: 33.1 ± 15.0 CLES = 76.3; p = 0.005**	R: 1.1 ± 0.1 L: 1.3 ± 0.3 CLES = 67.3; p = 0.094	R: 1139 ± 482 L: 1255 ± 465 CLES = 56.4; p = 0.545	R: 0.8 ± 0.2 L: 0.9 ± 0.3 CLES = 59.6; p = 0.471
15.0	R: 4.7 ± 1.0 L: 8.1 ± 3.9 CLES = 77.6; p = 0.005**	R: 14.9 ± 4.5 L: 28.3 ± 14.0 CLES = 78.2; p = 0.004**	R: 1.1 ± 0.1 L: 1.2 ± 0.3 CLES = 66.0; p = 0.201	R: 1053 ± 428 L: 1246 ± 368 CLES = 61.5; p = 0.236	R: 0.9 ± 0.3 L: 1.0 ± 0.2 CLES = 57.1; p = 0.689
17.5	R: 5.0 ± 1.2 L: 8.8 ± 4.4 CLES = 77.6; p = 0.005**	R: 16.1 ± 4.9 L: 26.9 ± 12.9 CLES = 77.6; p = 0.012*	R: 1.0 ± 0.1 L: 1.1 ± 0.3 CLES = 64.7; p = 0.308	R: 1222 ± 564 L: 1308 ± 417 CLES = 57.1; p = 0.665	R: 1.0 ± 0.3 L: 1.0 ± 0.3 CLES = 57.1; p = 0.610
OLS	Intercept: R: 2.4 ± 0.5 L: 4.8 ± 3.6 CLES = 76.3; p = 0.015*	Intercept: R: 25.5 ± 5.2 L: 48.2 ± 24.7 CLES = 75.6; p = 0.004**	Intercept: R: 1.2 ± 0.3 L: 1.7 ± 0.7 CLES = 74.4; p = 0.021*	Intercept: R: 736 ± 401 L: 915 ± 424 CLES = 64.1; p = 0.296	Intercept: R: 0.5 ± 0.2 L: 0.8 ± 0.3 CLES = 75.0; p = 0.042*
	Slope: R: 0.2 ± 0.1 L: 0.2 ± 0.1 CLES = 66.7; p = 0.112	Slope: R: − 0.6 ± 0.2 L: − 1.3 ± 0.8 CLES = 73.1; p = 0.010*	Slope: R: − 0.0 ± 0.0^a^ L: − 0.0 ± 0.0^a^ CLES = 76.9; p = 0.015*	Slope: R: 26 ± 34 L: 23 ± 24 CLES = 51.9; p = 0.801	Slope: R: 0.0 ± 0.0^a^ L: 0.0 ± 0.0^a^ CLES = 78.8; p = 0.009**

*CLES* Common Language Effect Size, *EPE* End Point Error, *IDE* Initial Direction Error, *PLR* Path Length Ratio, *RL* Response Latency, *PSR* Peak Speed Ratio, *R* Right Affected, *L* Left Affected, *OLS* Ordinary Least Squares

Significance level: *p ≤ 0.05, **p ≤ 0.01, and ***p ≤ 0.001

^a^PLR: Slope: R: − 0.011 ± 0.011; L: − 0.036 ± 0.034; PSR: Slope: R: 0.024 ± 0.008; L: 0.012 ± 0.012

### Interactions between speed and distance of the reference movement

**Fig. 4:**
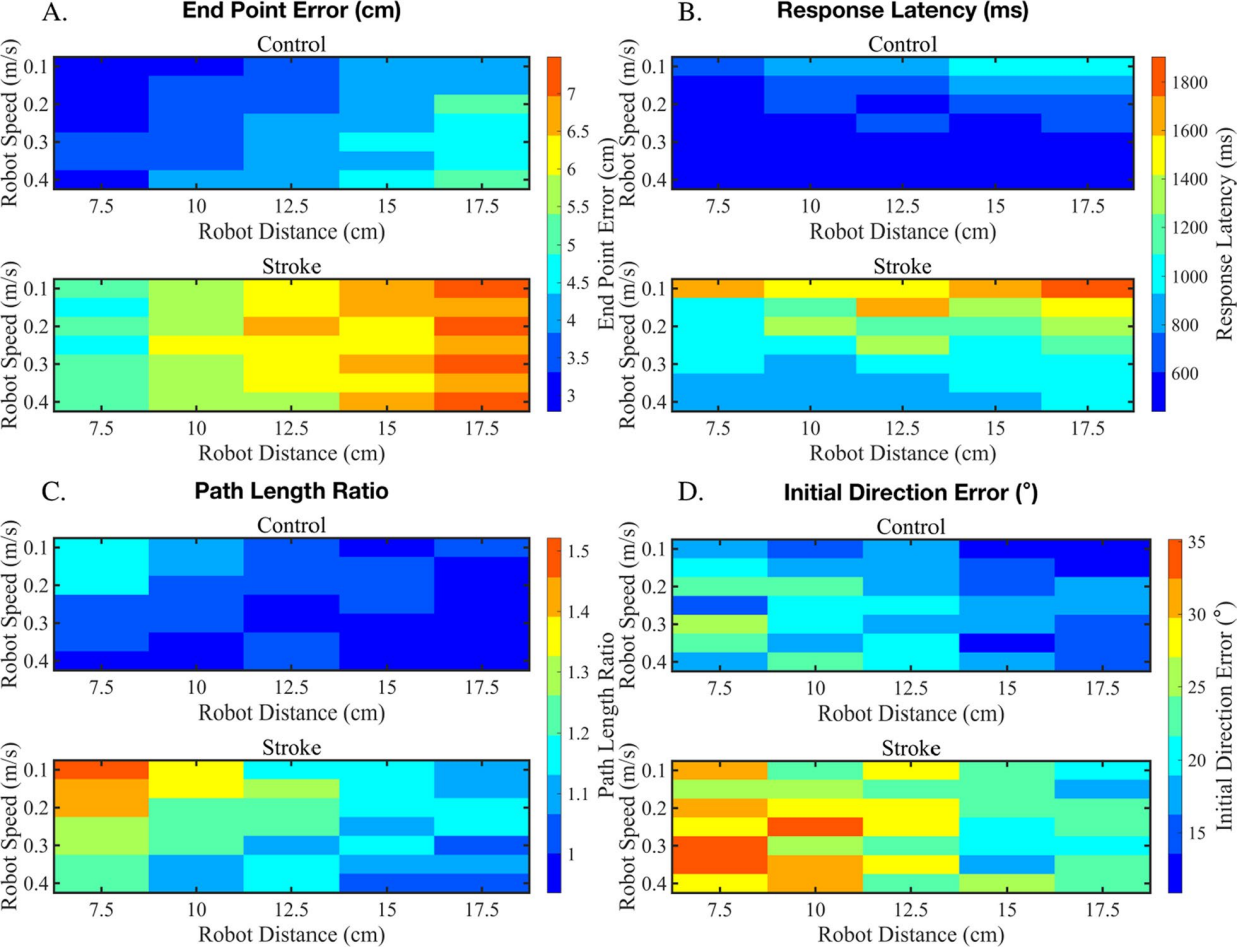
Heat plots demonstrating interaction effects for proprioceptive errors made with the active arm at a various combinations of speeds and distances generated by the passive, robotically-moved arm. In each panel, the color and value of the heatmap indicates the group level average for the respective proprioceptive outcome measure. We observed a significant interaction effect for Path Length Ratio (PLR, **C**) for both control (F = 10.66, p = 0.003) and stroke participants (F = 18.85, p < 0.001), and near significance for Response Latency in control participants (F = 4.00, p = 0.054). These interaction effects, particularly for PLR, suggest that participants systematically overshoot the target at low speeds and short distance combinations and undershoot reference movements that have fast speeds and long distance combinations

After analyzing proprioceptive error to specific reference speeds or distances, we aimed to determine whether there was an interaction for specific speed × distance combinations. For control participants, we used a two-way ANOVA (speed × distance) and found an interaction effect for PLR (Fig. 4C, top panel, F = 10.66, p = 0.003). Here, we observed that for PLR in controls, reference movements with slower speeds/shorter distances resulted in a larger amount of error. We completed the same analysis for the stroke group and found an interaction term also for PLR, which was considerably stronger than that observed for controls (Fig. 4C, bottom panel, F = 18.85, p < 0.001). These results suggest that as the reference movement becomes slower and shorter, the proprioceptive error related to movement length estimation increases considerably, suggesting distance perception in this range is much less accurate than other movement combinations, especially for individuals with stroke.

## Discussion

We found that stroke participants had significantly impaired proprioception compared to controls, regardless of speed or distance tested. Notably, we found that when we examined participants’ ability to match different reference speeds, stroke survivors regularly overestimated slow speeds and underestimated faster reference speeds. Further, we found that when participants matched different reference distances, stroke survivors had significantly worse performance at shorter distances, but that error lessened as the reference distance of the movement increased. These patterns suggest an amplified scaling of typical proprioceptive behavior observed in control participants.

Previous work has detailed that proprioceptive impairments are relatively common in stroke survivors and can significantly affect day-to-day function [2, 4, 6, 14]. The methodology used in prior studies to assess proprioception has typically focused on broad identification of proprioceptive impairments and has failed to survey reference movements of varying types. As such, it has only been in recent years that proprioception has been considered an important aspect of upper limb function after stroke, which means that we are only just beginning to understand how sensory information is impacted after stroke [42]. Methods that interrogate sensorimotor behavior at a deeper level, as is presented here, are critical to better understanding how proprioception is impacted after stroke, as activities of daily living require varied movement speed and distance that are dependent on task goals [43, 44]. The current study aims to build on previous work, in that the task design seeks to identify global impairments in limb proprioception, but also aims to detail whether the magnitude of these impairments is the same across reference speed or distance. Here, we test proprioceptive error across a broad range of speeds and distances to determine whether proprioception is uniformly or differentially affected depending on the characteristics of the reference movement. Information related to how sensory information is used or impacted across a wide breadth of movements will be informative to rehabilitation programs. An example of this is that it may indicate that daily functions with small movement distances (e.g., brushing your teeth) are differentially impaired compared to daily functions with large movement distances (e.g., reaching for a water glass).

### Scaled error responses to reference speed and distance in stroke survivors

The paradigm used in this study allows for insight into how individuals with stroke perceive movements with varying characteristics representative of daily life. Previous work has found that individuals with stroke have impaired perception of an “average” movement [4, 6, 45–47]. Overall, we observed that stroke participants had significantly impaired proprioception compared to control participants. Here, we found that stroke participants had impaired perception of movement regardless of the reference speed or distance tested. Our metrics showed that individuals with stroke consistently made significant errors for each movement speed and distance with increased End Point Error, increased Response Latency, poor estimation of speed (PSR) and distance (PLR) and increased Initial Direction Error compared to age-matched controls. These results demonstrate that these impairments are not the result of a singular type of movement, as has been previously shown, but that proprioceptive impairments affect a broad range of movement types after stroke.

While control participants showed some linear scaling of error responses for reference speed (e.g., End Point Error), we found that stroke participants had increased error across parameters compared to controls, and notably had amplified error scaling compared to controls (e.g., Peak Speed Ratio). Here, stroke participants tended to significantly overestimate slower speeds and underestimate faster speeds, but performed similarly to controls for speeds in the middle of the speed distribution (Fig. 2F). This suggests that stroke participants had more difficulty perceiving reference speeds at the tails of the testing distribution (e.g., 0.1 m/s, 0.4 m/s). We observed a similar pattern for reference distance; however, when we examined the ability of stroke survivors to match the length of the reference movement, we found that stroke survivors significantly overestimated the length of the movement when the distance was short (7.5 cm) and less so when the reference distance was longer (17.5 cm) (Fig. 3F). Previous work has shown that spindles remain relatively unaffected after stroke, and that it is likely that incoming afferent signals and outgoing efferent signals are poorly integrated [48, 49]. The fact that we observe differential responses to both reference speed and distance suggests that spindle encoding is flexible and may be optimized for detecting particular movement characteristics [50].

### Interaction between proprioceptive errors resulting from movement reference speed and distance

Surprisingly, we found few interactions between particular combinations of reference speed and distance. We observed a significant interaction effect for a single parameter, Path Length Ratio, where for both control and stroke participants, we observed that participants made significantly higher errors for movements that were both slower (e.g., 0.1 m/s) and shorter (e.g., 7.5 cm), suggesting that distance-based errors or perception may be particularly susceptible to estimations of both length and speed of the reference movement (Fig. 4C). Further, the lack of interaction effects observed in the remaining measurements suggests that proprioceptive performance for End Point Error, Initial Direction Error, Peak Speed Ratio, and Response Latency were dependent on either reference movement speed or distance, but not both. Interestingly, the remaining spatial measures, End Point Error and Initial Direction Error, appear to be more sensitive to changes in movement distance rather than movement speed for both groups (Fig. 4A, D). In contrast, temporal measures, Response Latency and Peak Speed Ratio, were more sensitive to changes in reference movement speed rather than distance for both groups (Fig. 4B). This difference may be due to the relative contributions of afferent fibers, where spatial parameters may be relying heavily on muscle length-based information relayed via Type Ia and II afferent fibers, where temporal information relies primarily on speed of muscle stretch via Type Ia fibers only [51]. Given that these patterns are generally preserved, but amplified in stroke participants, it is reasonable to assume that afferent information relayed to the brain is intact, and heightened errors occur as a result of poor translation of afferent information to efferent information [49].

### No notable effects of hemispheric lateralization of proprioception

For our analyses, we collapsed across the more affected side for individuals with stroke. However, previous work has suggested lateralization of proprioceptive information in stroke and individuals with sensory deafferentation [52, 53]. To determine whether there were any effects of hemisphere of stroke, we reanalyzed and compared the results of those who were more affected on the left side of the body vs. those who were right-affected. We found that left-affected and right-affected stroke participants were clinically comparable on the FIM, FMA, and PPB. Notably, our right-affected group had slightly lower MoCA scores than our left-affected group; however, we must note that this comparison was just under the level of significance. Surprisingly, we observed few differences when comparing robotic parameters (Tables 3 and 4), confirming previous results [6, 54], and suggesting that for passive movement of limb there are minimal effects of lateralization. We observed in this study that the most impaired participants tended to be those who are left affected, which is consistent with previous work [4, 6].

### Potential limitations

As described above, lateralization of proprioceptive function is an ongoing conversation and requires further investigation. A limitation of the current study is that we are typically unable to recruit individuals with large left hemisphere stroke due to the presence of aphasia that may hinder participant ability to understand task instruction. Future work aims to further investigate the idea of lateralization by designing tasks and task instructions that are accessible for those with conductive aphasia. Additionally, we must note that the sex of our participant sample was skewed. We tested twice as many male participants as female participants in the current study. While the effects of sex on proprioception are not definitive and several studies have found negative or mixed results [55–57], it is a source of potential confound.

## Conclusion

Proprioceptive impairment across reference speeds and distances was common in stroke participants. We found that after stroke, proprioceptive errors can readily change as a function of movement characteristics. A better understanding of how stroke survivors use proprioception to perceive and interact with their world has significant implications for designing personalized rehabilitation strategies that address within patient variability for impairments of the upper limb.

## Data Availability

The datasets used and/or analyzed during the current study are available from the corresponding author on reasonable request.

## Abbreviations

MoCA: Montreal Cognitive Assessment
EPE: End Point Error
IDE: Initial Direction Error
PLR: Path Length Ratio
RL: Response Latency
PSR: Peak Speed Ratio
CLES: Common Language Effect Size
FIM: Functional Independence Measure
FMA: Fugl-Meyer Assessment
PPB: Purdue Pegboard
BIT: Behavioral Inattention Test
